# The Financial Sustainability of Programs to Initiate Medications for Opioid Use Disorder in Emergency Department Settings

**DOI:** 10.1111/acem.70185

**Published:** 2025-11-05

**Authors:** Dominic Hodgkin, Cindy Parks Thomas, Margot T. Davis, Jennifer J. Wicks, Shelly F. Greenfield, Zachary F. Meisel, Constance M. Horgan

**Affiliations:** ^1^ Heller School for Social Policy and Management Brandeis University Waltham Massachusetts USA; ^2^ Harvard Medical School and McLean Hospital Waltham Massachusetts USA; ^3^ Perelman School of Medicine University of Pennsylvania and the Center for Health Economics of Treatment Interventions for Substance Use Disorder, HCV, and HIV Philadelphia Pennsylvania USA

## Abstract

**Background:**

The US is experiencing an epidemic of opioid misuse and mortality. Effective treatments are available, including medications for opioid use disorders (MOUD), but they are greatly underused due to a variety of barriers. In response, some US hospitals have established programs to identify emergency department (ED) patients with opioid use disorders (OUD) and begin treatment with MOUD (“ED induction”). For this model to be widely adopted, financial sustainability for hospitals is critical. Little is known about the financial aspects of ED‐based treatment models, including insurance billing and reimbursement.

**Objectives:**

Our study addressed the following questions about ED‐based induction of OUD treatment: (1) Which components of this model are billable to insurers? (2) How do hospitals fund the components that are not billable? (3) Does ED‐based induction generate savings that could help fund that service?

**Methods:**

We conducted a qualitative study, involving semi‐structured interviews with officials at selected US hospitals. Potential interviewees were identified using a snowball sampling approach. We conducted 12 interviews across 10 states, mostly with urban teaching hospitals.

**Results:**

Key findings include, (1) medication costs are often billable to insurers, but costs of key para‐professional staff like peer navigators are not, requiring the hospital to absorb their salaries. Even some billable costs are reimbursed at low rates which challenge sustainability. (2) To fund non‐billable components, hospitals typically rely on time‐limited grant funding, including the federal 340B drug rebate program. (3) Several interviewees anticipated cost savings to their hospitals from reduced use of ED services by patients who had no (or low‐paying) insurance.

**Discussion:**

These findings indicate that some hospitals are able to sustain ED‐based induction of MOUD using time‐limited grant funding. However, wider dissemination of this model will likely require more stable funding streams, such as Medicaid reimbursement, paying adequate rates, and coverage of personnel.

## 1 Introduction

The US is experiencing an epidemic of opioid misuse and mortality, with an estimated 54,743 overdose deaths involving opioids in 2024 [1]. Effective treatments are available, including medications for opioid use disorders (MOUD), but they are greatly underused due to a variety of financial and other barriers described further below. In response, experts are promoting the use of low‐barrier care in settings that routinely treat patients with opioid use disorder (OUD) [2]. One such setting is the hospital emergency department (ED), which regularly sees substantial numbers of patients with OUD, including overdoses [3]. In response, in recent years, some US hospitals have established programs in their emergency departments to identify ED patients with OUD and start them on MOUD (“ED induction”) [4]. These programs typically use peer specialists or support navigators who approach ED patients with OUD and offer to initiate them on buprenorphine, as well as connecting them with a post‐ED (e.g., “bridge”) program to continue OUD treatment [5, 6]. In 2017, the Substance Abuse and Mental Health Services Administration (SAMHSA) started allowing states to fund this model through grants from the Opioid State Targeted Response program [7].

Several studies, including a systematic review, have found hospital ED‐based MOUD programs to be effective at engaging patients in MOUD treatment after 30 days [8, 9, 10]. The programs have also been found cost‐effective from a societal perspective, with one study finding that at all positive willingness‐to‐pay values, ED‐initiated buprenorphine treatment was more cost‐effective than brief intervention or referral alone, using results from a randomized trial [11]. In 2021, the American College of Emergency Physicians endorsed this approach, recommending that emergency department physicians offer to initiate OUD treatment with buprenorphine in appropriate patients and provide direct linkage to ongoing treatment for patients with untreated OUD [12]. However, for MOUD induction to be widely adopted in emergency departments, it would need to be financially sustainable for the hospitals that operate those departments. Little is known about the financial aspects of ED‐based treatment models, including insurance billing and reimbursement issues, but prior research has identified some financial barriers. First, some model components, for example, peer support or navigator services, are not typically covered by insurers [13]. Second, although some other components are funded (e.g., through a new billing code issued by the Centers for Medicare and Medicaid Services in 2021) [14], the reimbursement rates are often very low [15]. Third, programs are often initiated using government grants, and rely heavily on them. While these grants are helpful, they are time‐limited, which reduces the predictability of funding [7, 16]. On the other hand, some studies have suggested potential financial benefits that operating an ED‐based MOUD program could achieve for a hospital, for example, savings from reduced use of inpatient addiction treatment in that hospital [17].

To help inform hospital officials and other stakeholders considering this model, our study addressed the following questions about ED‐based induction of OUD treatment: (1) Which components of this model are billable to insurers? (2) How do hospitals fund the components that are not billable? (3) Does ED‐based induction generate savings that could help fund that service?

## 2 Methods

We conducted a qualitative study, involving semi‐structured interviews with officials and clinicians at selected US hospitals, and with experts. Our methodology was based on thematic analysis. Our reporting of the study methods adheres to the Consolidated Criteria for Reporting Qualitative Research (COREQ) [18].

### 2.1 Conceptual Framework

Our framework for this study starts from an economic model of how adding an ED‐based MOUD program affects a hospital's financial position, adapting our earlier related work [19]. Assuming that the new program would require providing a monthly volume of billable services (*q*_b_), which are reimbursed at *f*_b_ per service, the hospital would also have to deliver other services that are not billable (*q*_u_). The hospital's operating costs (*C*) depend on its volumes of both *q*_b_ and *q*_u_, while its revenues come both from reimbursement of billable services and from grants (*G*) that are not tied to service volume. Thus, the hospital's operating surplus (revenues minus costs) is given by:

$$\text{Operating surplus}=f_{b}q_{b}+G-C\left(q_{b},q_{u}\right)$$

Adding a new activity will only be sustainable if the operating revenues at least cover the operating costs. The delivery of non‐billable services will thus need to be covered from other sources, such as grant revenue, or cross‐subsidization from any surplus revenue from billable services, or both. Another possibility is that adding the activity reduces the unit cost of delivering other services. If the grants and/or the payment rates for billable services are too low, and if no cost savings result, then the new activity may not be sustainable.

A more complete model would need to consider other issues such as the cost of any initial investment (including training navigators), administrative costs, and the existence of multiple services and multiple payers with different reimbursement rates. Nonetheless, this framework serves to motivate our three study questions, with their focus on the key distinction between billable and nonbillable services.

### 2.2 Participant Recruitment

We identified potential interviewees using a snowball sampling approach, starting from individuals who had published articles about relevant programs at their ED, or were otherwise known to the research team, or through our professional networks. We targeted clinicians and administrators, anticipating that they were the most likely to know about financing issues. We emailed selected individuals an invitation up to 2 times (two weeks apart), and offered $50 to compensate for their time. We informed participants that the study goal was to identify the financial issues affecting whether it is financially sustainable to implement induction of MOUD in a hospital ED, and that the interviewers were academic researchers interested in the viability of this model.

### 2.3 Interview Guide

We developed a semi‐structured interview guide that reflected our study questions (see Appendix A). We also developed a list of deductive codes based in part on our conceptual framework described above, which included: Barriers, billing insurers, effectiveness, facilitators, financial viability, grant funding, medication costs, peer staffing, savings, service design, staffing, and sustainability. This guide was not provided to interviewees beforehand, but our invitation email indicated our general focus.

Interviews were led by authors D.H., C.P.T., and M.T.D., with participation from authors J.J.W. and C.M.H., all of whom are health services researchers with doctoral degrees, and S.F.G. who is a physician. Z.F.M. is a physician. All authors had experience conducting clinician interviews. C.P.T., M.T.D., J.J.W., S.F.G., and C.M.H. are female, and D.H. and Z.F.M. are male. All interviews were 1 h or less, and all were videorecorded and redacted. No one attended the interviews other than the participants and researchers. Interviewing continued until data saturation was reached. No repeat interviews were conducted. Transcripts were not shown to participants, nor were they asked for feedback on the findings. The research protocol was approved by the institutional review board at Brandeis University, and all interviewees provided informed consent verbally. No large language model was used to write any part of the submission.

### 2.4 Qualitative Analysis

To develop the analytic frame, the authors conducted three informational interviews with experts on the topic of ED‐based induction of MOUD. Qualitative analyses were conducted by two separate coders, using the deductive code list and Atlas ti.24 software. The coders reviewed initial coding together to ensure alignment of their coding applications, and reconciled discrepancies, before completing all transcripts. Once all transcripts were coded by both coders, they used the quotation reports generated by the software to develop themes to compare within and across hospital categories. The coders developed summary reports to aggregate the data and reviewed each other's findings.

### 2.5 Study Sample

Our data collection process resulted in 12 interviews across 10 US states, including sites in all four census regions (Northeast, Midwest, South, West). Some interviews included multiple staff, yielding a total of 18 interviewees, and several staff occupied multiple roles. Although most interviewees were ED‐based clinicians located at urban teaching hospitals, other roles included emergency medicine pharmacist, psychiatrist, case manager for MOUD (sometimes called “medication assisted treatment” (MAT)), doctoral student, registered nurse, patient safety specialist, quality improvement personnel, vice president of finance, and revenue cycle manager. Several of these interviewees were emergency medicine chiefs and/or designed the ED induction protocols for their hospitals or systems (see Appendix B). Nine of the interviewees were female and nine were male. We did not ask interviewees about their race or ethnicity. Only one interviewee was previously known to members of the study team. Seventeen individuals did not respond to invitations, and two declined the invitation.

## 3 Results

Detailed results are reported below by theme, and the barriers to and facilitators of implementation and sustainability are summarized in Table 1.

**TABLE 1:** Key barriers to and facilitators of implementation and sustainability of ED induction.

Barriers	Facilitators
Low reimbursement rates from insurers, particularly for peer and consulting services High cost of brand name or injectable MOUD that are not reimbursed Insurers only covering medication costs through a pharmacy, not hospital Reliance on and loss of short‐term grant funding for services (e.g., telehealth, navigators that facilitate follow‐up care) 340B programs only covering medications for inpatient Difficulties gaining hospital buy‐in to cover service costs	Dedicated ED SUD service rather than consulting service Providing HIV/Hepatitis C screening and treatment, which are reimbursable Partnering with pharmacies to facilitate patient medication access Grant funding to cover medications for uninsured, navigators, and services to address social determinants of health (e.g., transportation) Navigators, peer coaches, bridge clinics, and telehealth services to support follow‐up care 340B discounts for MOUD administered in ED and prescriptions filled in hospital pharmacy Using data, revenue projections, and tracking follow‐ups/readmissions to demonstrate effectiveness and gain hospital buy‐in

### 3.1 Financial Viability and Sustainability

Interviewees reported that hospitals use multiple external sources (grants, third‐party payers, and incentives) and internal sources of funding to support their ED induction programs. Several interviewees commented that there are minimal additional costs for providing induction. The doctors are already there, buprenorphine does not cost much, and is “cost neutral” (ID 12b) for some because they already had systems in place. One said “The doctors are mine. The guidelines are mine. So really the only cost is the follow‐up setting” (ID 11). However, some of the same interviewees, as well as others, expressed concerns about keeping the program running long‐term, particularly to support follow‐up care. One interviewee said “That's a big question. Once the grant ends, how are we going to keep this going…we help with transportations to appointments after they leave the ER. We help cover prescriptions for buprenorphine. The buprenorphine that we administer in the ED just gets rolled into their emergency department bill, but then there's a whole other step. They need to have this prescription until we can get them to their appointment” (ID 8), highlighting costs of transitioning patients to follow‐up care and the need for financial support to sustain care. One interviewee said, “I think people have to understand…I mean, you have to have a sustainable model, you can't just live off hopes and dreams (ID 2)”.

#### 3.1.1 Grant Funding

Most interviewees said they currently rely on grant and/or philanthropic funding. Often the ED program staff applied for the grants, but sometimes hospital administrators or other partners applied for grants as well. Some interviewees expressed concern that when the grants end, the hospitals will not know how to keep the program running. One interviewee said it is “rare that an ED is going to break even. There's always some subsidy going there as well” (ID 7). Often hospital administrators want to see program effectiveness or means for generating revenue prior to providing financial support. Another interviewee commented that “we cannot make a financial case, just that we are providing care to vulnerable populations (ID 6a)”. ED clinicians in our sample hoped to prove that ED induction is an effective medical practice during the grant period.

Grant funding is primarily used to support staff, including physicians, nurses, navigators, peer coaches and counselors, either in the ED or in aftercare clinics. Telehealth services offered by ED aftercare staff are also largely grant‐funded, as insurers only reimburse telehealth delivered by credentialed providers. Grant funding may also be used for medication, including naloxone, administered during the ED visit. Grant funding is particularly helpful to cover the costs of the uninsured.

Grant funding sources ranged from federal (e.g., SAMHSA, NIH) and state (e.g., opioid settlement) to specific institutions, programs (e.g., an overdose prevention network), or companies. Grant funding is typically focused on MOUD and targeted at funding nonbillable costs such as peer support, and less often supports evaluation activities.

#### 3.1.2 Third Party Payers

Interviewees said that having a variety of payers is important for ED induction viability and sustainability, and that EDs need commercial insurers in the payer mix. Because Medicaid is managed at the state level, we found differences across EDs in what Medicaid covers. For example, not all Medicaid programs pay for in‐clinic administration of MOUD, including injectable extended‐release versions of naltrexone and buprenorphine. Most interviewees reported that their state Medicaid program currently does not reimburse at a high enough rate to meet the costs of ED induction (including staff time). Commercial insurance usually covers the costs of MOUD prescribed in the ED, but interviewees said that only a small percentage of their patients with OUD were covered by commercial payers.

Many EDs routinely screen patients with OUD for human immunodeficiency virus (HIV) and hepatitis C. Both the screening and treatment for the conditions are billable and the revenues help offset costs of induction. Another interviewee said that insurance reimbursement for Screening, Brief Intervention and Referral to Treatment (SBIRT) may help cover the costs of ED induction as well.

Interviewees said that the type of MOUD prescriber and patients' point of entry determine the costs to the hospital. For example, providing ED induction through an addiction consultation model, where the prescriber is located in a non‐ED medical or psychiatric unit [20], brings the hospital less revenue because the consulting services are generally billed at a lower rate than for a dedicated ED addiction medicine team. Conversely, with some payers, when MOUD is administered during the ED visit, the medication is included in the bundled rate (where a single payment covers all services related to a specific episode of care), but if the medication is administered in an outpatient clinic, the hospital can bill separately for it.

Some interviewees talked about their partnerships with specialty pharmacies as a critical piece of sustainability. Specialty pharmacies are located in the hospital or in the community and stock MOUD medications. These partnerships “can reduce cost by supporting patient access to medication and reducing repeat ED visits due to medication issues (ID 5)”. However, not all pharmacies accept Medicaid insurance, so linking patients to the right pharmacy is an important piece of aftercare.

#### 3.1.3 Incentives

Some states' Medicaid programs offer incentives for the ED to write the prescription for MOUD, and for follow‐up care. Interviewees in one state said Medicaid has incentive programs for seeing people with behavioral health concerns and this can provide additional financial stability. Some insurers provide incentive payments to programs that meet performance targets for linking people with behavioral health diagnoses to care. One measure used for incentive payments is the proportion of patients who are linked to outpatient care within 30 days after their emergency care visit. Another interviewee reported that payments from the state incentive program go to the hospital corporate office, which can limit the program's ability to access the funding directly for services supporting ED induction.

#### 3.1.4 Indirect Funding

Many interviewees said that in calculating costs and benefits of ED induction, they anticipate that the hospital may lose money for the ED visit, but that the high rate of aftercare visits in their outpatient department will offset ED costs. Some programs derive partial funding from the 340B drug rebate program, which allows safety‐net facilities to purchase medications at a steep discount (25%–50%). The ED may then charge insurers the non‐discount price, and use the resulting margin to fund health care [21]. One interviewee said that the 340B program is not a big source of income, due to the low cost of generic forms of MOUD even before discounts (e.g., $0.34 per tablet for buprenorphine). Also, some hospitals pass savings from 340B along to the patient, especially the uninsured. However, extended‐release injectable non‐generic versions of MOUD are more expensive, for example, $3000 per month for buprenorphine, and various interviewees reported the 340B revenue as significant. One hospital's social work department has an emergency fund that the ED can access, for patients who cannot afford the medication. Another hospital has a charity care budget for the uninsured.

Interviewees said they want to remove as many barriers to ED induction as they can, but the timing of the patient visit is a factor, especially weekends and holidays. Some prescribers are hesitant to give a 3‐day prescription of buprenorphine until the patient can visit an aftercare program, due to the potential of misuse. On the other hand one hospital gives take‐home buprenorphine‐naloxone kits at no cost to patients, which they say has increased the follow‐up rate from 41% to 83%. The same hospital attempts to administer injectable MOUD if it is clinically appropriate.

Having a critical volume of patients is key to viability and sustainability, and that requires being located in an area with a sufficiently large population seeking services for OUD. Some interviewees talked about the longer‐term effect of ED induction, which is that it “lowers repeat ED visits, which the hospital wants” (ID 1).

### 3.2 Effectiveness and Savings Generated

Interviewees discussed how hospitals think about the effectiveness of ED induction and how they generate savings. One interviewee commented, “We can start to show some benefits to the hospital, that we can bring something else to the table…There's a real benefit outside of just the social aspect and getting patients to the next level in their care. That we can actually impact it on a financial bottom line. Healthcare systems always need to see that (ID 7)”. One interviewee said their ED induction program is self‐supporting because they do get a return flow of revenue through insurance reimbursement. Some EDs create business plans, focusing on the value of follow‐up care, reducing overdoses, and reducing ED readmissions, to determine the best revenue projections for treating OUD in the ED or in satellite programs.

Interviewees talked about ways that administering MOUD in the ED provides clinically appropriate care and reduces recidivism, a financial burden for hospitals. Prescribers include RNs and LPNs who are paid a lower rate than physicians in the ED. If the ED has a partnership with a pharmacy, the patient faces less of a barrier to filling a prescription, which reduces recidivism. Patients inducted in the ED reportedly have high attendance rates at outpatient and bridge clinics. One hospital has a tracking system that shows that 87% of patients entering the ED with OUD attended their follow‐up appointment. This hospital also compares data pre and post ED induction implementation that shows that MOUD administered in the ED reduces length of stay, especially for patients with co‐occurring psychiatric diagnoses. Another interviewee from an urban teaching hospital said it is hard to track patients post‐induction, but to facilitate follow‐up, some EDs give patients enough MOUD to bridge the gap before outpatient care, rather than providing a prescription that must be filled in the pharmacy.

### 3.3 Service Design and Staffing

Generally, the protocol for ED induction was similar across our sample. Once the patient is diagnosed with OUD, they receive counseling on treatment options from a physician or other staff member. If the patient agrees to MOUD, they are given a dose at the hospital, held for observation, and given a prescription for buprenorphine. Patients who prefer methadone are linked to an Opioid Treatment Program (OTP). All EDs in our sample use buprenorphine and one additionally utilizes methadone. Some EDs have a bridge clinic where they follow a patient until outpatient care is available. “Bridging” a patient to follow‐up care is often done through telehealth in teaching and community hospitals. If the patient's pharmacy is closed for the weekend, the ED physician will give three days of medication to continue the treatment. Some EDs offer transportation to the pharmacy. In some hospitals the physician will assess if a patient is appropriate for MOUD, and then the registered nurse or licensed practical nurse provides the prescription.

Interviewees said that the number of patients the ED can treat for OUD has increased since 2022, when the federal government eliminated the X‐waiver requirement (a policy restricting who could prescribe buprenorphine after the first 3 days). An interviewee from a rural hospital said they have challenges recruiting professional staff to the ED. Non‐clinical staff in EDs (e.g., navigators, peer coaches) provide valuable work helping the patient locate a pharmacy to fill the prescription and find aftercare, but interviewees noted low payment for those staff. Most interviewees said those positions are funded through grants because many of the state Medicaid programs will not reimburse services from para‐professional staff. ED staff perceive that since persons with OUD generally are not engaged in outpatient behavioral health or medical systems, ED induction is an appropriate and necessary intervention. Interviewees said there is support for ED induction among physicians and administrators. In one state a regulation requires that the first appointment for a controlled substance must be in‐person. This creates a dilemma for ED physicians, who are hesitant to induct a patient knowing that it may be months before they can see an outpatient provider.

ED clinicians try to keep the patient within their system for continuity of care and encourage patients to obtain MOUD from specialty pharmacies within the hospital system. Some interviewees said their hospital is actively engaging in outreach to locate individuals with OUD. One works with jails and community organizations to identify people needing services, and another surveyed Latinx outreach workers to develop a program. Often ED doctors routinely screen for hepatitis C and HIV, because those conditions are often correlated with OUD, also involving syringe use.

#### 3.3.1 Peer Staffing

Peer navigators, also called recovery coaches and peer coaches, work in every ED where we interviewed, except in two teaching hospitals. Their roles vary. In one ED, the peer navigator checks which pharmacies take the patient's insurance and stock MOUD. The navigator also arranges transportation for follow‐up appointments. This hospital said navigators have a caseload of up to 300 patients a year. Other tasks of the navigators include referring patients to rehabilitation clinics and working with prescribers to write a bridge prescription until access to the next level of care is secured. They engage patients in harm reduction education, send patient information to referral sources, and coordinate all aftercare. In one hospital the navigator is located in the aftercare clinic and when called by ED staff, will walk the patient to the aftercare clinic for a warm hand‐off.

Prescribers said they like to work with navigators because now they can refer patients with OUD to care, and can track the prevalence of OUD in their communities. Navigators are also valued because it is estimated by one hospital that they help reduce ED admissions “five‐fold” (ID 4) and steer patients into primary care instead of the ED.

## 4 Discussion

In this qualitative study of ED induction programs, we found that hospitals were able to implement programs using a variety of funding approaches, but continued to face challenges around long‐term sustainability. Below we relate these findings to prior research and the broader context, organizing the discussion around our research questions.

Our first question concerned which of the model's key components could be billed to insurers. We found that medication costs were typically billable, but the costs of key para‐professional staff like peer navigators were not. This confirmed a number of earlier reports about the inability to bill key staff [4, 5, 22]. In 2021, Medicare introduced a new billing code for “Initiation of medication for the treatment of opioid use disorder in the emergency department setting” (G2213), and various state Medicaid programs have adopted it [14]. However, this code can only be billed by physicians, physician assistants and registered nurse practitioners. Prior studies had noted other types of induction‐related costs that hospitals could not bill for, such as project manager support or data system support [23]. A related problem we found is that even if a service is billable, the payment rate may be so low as to challenge sustainability. This issue has also been reported in one state, where the state's Medicaid program started paying for peer recovery coaches, but hospitals did not consider the reimbursement to be adequate for sustaining ED induction efforts [15].

The second question was how hospitals were financing the costs of non‐billable services. We found that hospitals are typically relying on time‐limited grant funding, including state grants and the federal 340B drug rebate program. Hospitals also rely on billing for related activities, for example, HIV/hepatitis screening. Prior research has discussed most of these funding sources [15, 22]. One exception is the 340B program, which has proved helpful at hospitals eligible to access it, although it may be scaled back under the new federal administration. Another strategy mentioned in prior literature was to expand the ED addiction program to treat substances beyond opioids (particularly alcohol) [5], which can provide additional patient volume to justify hiring a navigator. In addition, some payers are introducing bundled payment for these services, which potentially gives hospitals the option to use more paraprofessionals without having to worry about the ability to bill their services [4].

Our final question was whether ED induction generates savings for the hospital that can help fund the service. Some hospitals we interviewed anticipated cost savings from reduced use of ED services by patients with severe disorders who had no (or low‐paying) insurance. Similarly, additional visits to the bridge and hospital‐based outpatient clinics generate revenue for the hospital that may help offset other costs. Prior research has occasionally hypothesized such savings [8], but they have not been measured to date.

Of note, when evaluators surveyed the 52 hospitals funded by California's CA Bridge state grants in 2020, most expected to continue to treat ED patients with buprenorphine after the end of that funding, although only 36% of hospitals reported that they would continue providing the full array of CA Bridge services. The evaluators noted that CA Bridge was intended to support hospitals during the start‐up period with intensive technical assistance and financial support, but that after the intervention period, costs should be significantly lower [24]. This highlights the wider issue that different factors may influence initial implementation versus ongoing financial sustainability of ED induction.

ED induction of MOUD faces some emerging issues beyond what interviewees raised. First, a growing proportion of patients arrive at the ED after overdosing on fentanyl, which creates concerns among clinicians and patients about the potential risks of precipitated withdrawal from starting buprenorphine [25]. As a result, some EDs also dispense methadone for a few days until such patients can be connected to an OTP. Second, some health systems have started programs where personnel responding to emergencies in the community (e.g., ambulance staff) offer overdose patients buprenorphine before they are transported to the ED [26]. This also helps those patients who refuse to be transported to the ED, but reportedly it is not yet common practice. Third, proposed changes to Medicaid and federal grant funding could pose major challenges to the ED induction approach.

More broadly, financial sustainability is only part of the wider context affecting the sustainability of ED induction, albeit an important part. Other important implementation issues include the readiness of hospitals to start programs, the presence of champions, the role of external policies, and support for MOUD in the community.

### 4.1 Study Limitations

The main limitation of this study is a lack of information about hospitals that decided not to adopt an ED‐based MOUD induction program, or that started one and then stopped it. This limits our information about barriers to program adoption, but is aligned with our focus on sustainability of programs. A second limitation is that our sample mostly comprised urban teaching hospitals, potentially reducing generalizability to other settings such as small, rural, and/or non‐teaching hospitals.

### 4.2 Implications

For hospital decision makers, our study suggests that despite financial challenges, ED induction of MOUD appears to be economically feasible in at least some hospitals, particularly large urban ones. The service may appear loss‐making if considered only from the ED perspective, but may save costs elsewhere in the hospital system (an “offset”; as is likely also true for some other services that are started in the ED, such as injury treatment or cardiovascular care). For wider future dissemination, it might be helpful to provide hospitals with a standardized tool to evaluate the likely financial impact of adopting this program, including what specific revenue sources and cost issues they should investigate. Some resources have been developed along these lines [17], but more specificity would be helpful to inform hospital decision‐making. In particular, rural hospitals may face special challenges with this model.

For policymakers, a key implication is the need to promote more stable funding streams, to allow hospitals to recruit and retain trained staff. The simplest approach would be to include this model in the standard sets of services covered by private and public insurers, as has been occurring for some other novel delivery models [27]. This funding would need to support services that are currently non‐billable such as those provided by para‐professional staff, at payment rates that cover costs. Greater use of alternative payment models such as bundled payment could also be helpful in giving hospitals more flexibility about what staff to deploy. All these funding sources may face future challenges due to proposed changes to Medicaid and federal grant funding. Funding challenges may lead policymakers to question the need to treat MOUD specifically in the ED, considering that it is one of the costliest health care settings. However, since the ED is where many patients with OUD present for care, it aligns with good medical care to start treating the problem there using an evidence‐based approach, while planning for a transition to less costly outpatient settings.

## 5 Conclusion

This study finds that hospitals face substantial challenges in sustaining ED induction programs, including the inability to bill for key components of the model. However, some hospitals see this as a critical service, and are able to sustain these programs through the use of a variety of temporary funding sources. Some hospitals also anticipate cost savings that will partly pay for the ED induction programs. Wider dissemination of this program will require a transition to more reliable long‐term funding, such as full coverage by insurers.

## Author Contributions

D.H. and C.P.T. developed the study concept and design. D.H., C.P.T., and M.T.D. led the interviews with participants, using interview guides developed with input from C.M.H. and S.F.G. M.T.D. and J.J.W. conducted the qualitative coding and wrote the results section. D.H. drafted the introduction and discussion sections. All authors participated in the critical revision of the manuscript for important intellectual content.

## Conflicts of Interest

The authors declare no conflicts of interest.

## Data Availability

Research data is not shared. The data are interviews with hospital officials who were promised confidentiality as part of consent.

## Appendix A Interview Guide

1. Please briefly describe your current job, and your involvement with SUD treatment in the ED.
2. For interviewees who work for a hospital that already has an ED induction program:
  - What were key influences on your hospital's decision to add this program?
  - What are the most important variables affecting its viability? E.g., the proportion of ED visits involving OUD; cost per hour of labor (ED physicians; peer support; other). (Note: we will focus on quantifiable variables.)
  - What are the current values of those variables at your hospital, e.g., the proportion of ED visits that involve OUD?
  - What are key aspects of your program's design that are relevant to its financial impact? (E.g., use of a bridge clinic; use of hospital‐based staff versus community‐based staff; receiving any grant funding)
  - Does your program address other substance use disorders besides OUD?
  - What approaches might make this model easier versus more difficult for a hospital to adopt? (E.g., use of a bridge clinic; use of hospital‐based staff versus community‐based staff)
  - Have you seen or done any financial modeling of a program like this?
3. For interviewees who work for a hospital that does not have an ED induction program:
  - Has your hospital considered adopting this program?
  - If yes, what were key influences on your hospital's decision not to adopt it?
  - What were the most important variables affecting its viability? E.g., proportion of ED visits involving OUD; cost per hour of labor (ED physicians; peer support; other). (Note: we will focus on quantifiable variables.)
  - What are the current values of those variables at your hospital, e.g., the proportion of ED visits that involve OUD?
  - What approaches might make this model easier versus more difficult for a hospital to adopt? (E.g., use of a bridge clinic; use of hospital‐based staff versus community‐based)
  - Have you seen or done any financial modeling of a program like this?

## Appendix B Interviewee Information

**Table:**

ID	Region	Urban/Rural	Hospital type	Roles
1	NE	Urban	Teaching	Patient safety specialist, QI, lead emergency medicine pharmacist
2	S	Urban	Teaching	Emergency medicine, medical toxicology, and addiction MD; emergency medicine faculty
3a	NE	Urban	Teaching	Emergency department MD, ED induction protocol development, emergency medicine faculty, director of research/community engagement and emergency department health education programs
3b	NE	Urban	Teaching	Addiction medicine psychiatrist
4a	W	Urban, Rural	Community	PA, hospital system and ED induction design, bridge program founder
4b	W	Urban	Community	PhD, biomedical researcher, patient care, electronic medical record management, work flow and protocol development
5	S	Urban	Teaching	VP of finance in university health care system, ED finance
6a	NE	Urban	Teaching	Emergency MD, director of addiction medicine in university health care system
6b	NE	Urban	Teaching	ED addiction medicine revenue cycle manager
6c	NE	Urban	Teaching	Director of ED revenue cycle management
7	MW	Urban	Teaching	Chief of emergency medicine/medical director
8	S	Urban	Teaching	Emergency medicine MD and faculty
9	W	Urban	Teaching	Emergency and addiction medicine MD and faculty
10a	NE	Urban	Teaching	Addiction MD and medical director of addiction services
10b	NE	Urban	Teaching	Addiction medicine psychiatrist
11	NE	Urban	Teaching	Chair of university emergency medicine; emergency medicine, toxicology, and addiction medicine MD; chair of pharmacy and therapeutics committee, protocol development
12a	W	Rural	Community	Clinical project manager, nurse, MOUD program lead in family medicine and ED
12b	W	Rural	Community	Part‐time ED nurse, MOUD case manager, doctoral NP student

Abbreviations: MW, Midwest, NE, Northeast, S, South, W, West.
